# Perceptions and Willingness of Physiotherapists in India to Use Telerehabilitation During the COVID-19 Pandemic

**DOI:** 10.5195/ijt.2021.6425

**Published:** 2021-12-16

**Authors:** Arnold Fredrick D'Souza, Sydney Roshan Rebello

**Affiliations:** 1 Department of Physiotherapy, Father Muller College of Allied Health Sciences, Mangaluru, India

**Keywords:** COVID-19 Pandemic, Perception, Physiotherapists, Telehealth, Telerehabilitation

## Abstract

The COVID-19 pandemic and ensuing lockdowns have restricted regular clinical physiotherapy services. This has necessitated a sudden shift to the use of telerehabilitation to prevent disruption in the delivery of physiotherapy interventions. This survey investigates the perceptions of physiotherapists in India and their willingness to use telerehabilitation during the pandemic. An electronic questionnaire was sent to 176 physiotherapists around India, and 118 completed questionnaires were received (acceptance rate of 67.04%). A majority of the respondents (n=67; 77%) had used telerehabilitation for the first time during the pandemic, and 72.9% (n=86) found telerehabilitation to be a viable option for healthcare delivery during the pandemic. Some of the barriers identified were lack of training (n=64; 52%) and a lack of connection between information and communication technology experts and clinicians (n=62; 52.5%). Overall, physiotherapists in India expressed a positive perception of telerehabilitation and are willing to use such services.

The COVID-19 pandemic that began in early 2020 has had a significant impact on healthcare. Most governments worldwide enforced stringent lockdowns to curb the spread of COVID-19 (Barach et al., 2020). A sudden halt in in-person physiotherapy services prompted physiotherapists to find innovative solutions to remotely meet the needs of patients (Minghelli et al., 2020). Travel restrictions and fear of infection kept patients with rehabilitation needs away from clinics and hospitals (de Biase et al., 2020). Thus, telerehabilitation soon became a necessity.

Telerehabilitation, a branch of telemedicine, is defined as using Information and Communication Technologies (ICT) to provide rehabilitation services to people remotely in their homes or other environments (Brennan et al., 2009). The practice of telerehabilitation has been documented in the literature for over two decades. With advancements in technology and the ubiquitous nature of personal computing, providing telerehabilitation has become more accessible, and more clinicians and patients are opting for this model of healthcare delivery (Peretti et al., 2017). Studies have found telerehabilitation to be just as effective in various physiotherapy specialties as traditional in-person rehabilitation. Additionally, it is an economical alternative for patients residing in remote locations (Seron et al., 2021). The Centre for Health Exercise & Sports Medicine, University of Melbourne, has developed an evidence summary for physiotherapy care via telerehabilitation (Hinman et al., 2020).

International physiotherapy organizations such as the World Confederation for Physical Therapy (WCPT), now known as World Physiotherapy, American Physical Therapy Association (APTA), Chartered Society of Physiotherapy (CSP), and the International Network of Physiotherapy Regulatory Authorities (INPTRA) recommend the use of telerehabilitation (APTA, 2020; CSP, 2020; WCPT, 2020b, 2020a). World Physiotherapy has heavily promoted the use of telerehabilitation and has developed a task force to develop and disseminate resources on the subject (WCPT, 2020a). National physiotherapy bodies such as the Australian Physiotherapy Association (APA, 2020) and the Irish Society of Chartered Physiotherapists (ISCP, 2020) have already developed guidelines and policies for telerehabilitation practice within their respective countries. The International Private Physical Therapy Association, a subgroup of World Physiotherapy, surveyed its member countries and showed that digital physiotherapy practice had been steadily approved, utilized, and regulated since the start of the pandemic (WCPT, 2020a). India is not a member country, and to date, no survey has been conducted to address this knowledge gap.

Compared to the rest of the developed world, the implementation of telerehabilitation in India and other developing nations needs more research. India is culturally, socioeconomically, and geographically diverse, which poses a considerable challenge to creating uniform guidelines and policies suitable for every region (Shenoy & Shenoy, 2018). Presently, there are no guidelines for the benefit of telerehabilitation by physiotherapists in India. Clinicians have to resort to adapting guidelines prepared by international organizations to fit their needs. This survey aims to investigate the perceptions and willingness of Indian physiotherapists to implement telerehabilitation in their clinical practice. The results from the study may help inform policy-makers and stakeholders about the readiness of physiotherapists to adopt telerehabilitation. Additionally, the results from this survey may help researchers design telerehabilitation guidelines that are specifically tailored to the unique needs of physiotherapists practicing in India.

## MATERIALS AND METHODS

### ETHICAL CONSIDERATIONS

This study was approved by the Father Muller Institutional Ethics Committee (FMIEC/CCM/528/2021), and the protocol was registered in the Clinical Trials Registry - India (CTRI/2021/07/034666). All respondents provided written informed consent and were notified that their participation in this study was voluntary and could withdraw at any time.

### PARTICIPANTS

The convenience sampling method was used, and a sample size of 176 was obtained. The questionnaire was distributed through WhatsApp and electronic mail to physiotherapists around India with at least one year of clinical work experience. Reminders were sent to the physiotherapists who had not responded after a week. Data collection was performed over two months. The respondents' data was collected anonymously. This was specified to the respondents through a confidentiality statement mentioned within the survey. Of those surveyed, 118 respondents (acceptance rate of 67.04%) participated in the study and completed the questionnaire.

### QUESTIONNAIRE

A self-report questionnaire utilized in a previous study (Albahrouh & Buabbas, 2021) was adapted for use in this survey with the permission of the authors. The questionnaire was comprised of several sections: demographic data, technological background, perceptions of telerehabilitation, comfort with technology, willingness to use telerehabilitation, and barriers to using telerehabilitation. Responses for the last three sections were collected using a four-point Likert scale (from strongly disagree to strongly agree).

### DATA ANALYSIS

Descriptive data analysis was conducted. Frequencies and percentages were calculated for the respondents' demographic data.

## RESULTS

### PARTICIPANT PROFILE

Table 1 shows the demographic data of all respondents. A majority of the respondents under 35 years of age (n=81; 68.6%), held postgraduate degrees (n=81; 68.6%), specializing in the area of musculoskeletal physiotherapy (n=50; 42.4%), with less than 5 years of work experience (n=68; 57.6%), and practicing in urban regions (n=81; 71.3%). Most respondents were from the southern states of India (n=82; 73.7%) and employed in teaching hospitals (n=58; 49.2%).

**Table 1:** Demographic Data

Demographic variables	Number (%)
**Gender**	
Male	52 (41.1)
Female	65 (55.1)
Prefer not to say	1 (0.8)
**Age**	
Under 35	81 (68.6)
35 - 50	35 (29.7)
51 - 60	2 (1.7)
Above 60	-
**State/Union territory**	
Karnataka	55 (46.6)
Kerala	20 (16.9)
Maharashtra	14 (11.9)
Tamil Nadu	12 (10.2)
Goa	5 (4.2)
Gujarat	3 (2.5)
Delhi	2 (1.7)
Uttar Pradesh	2 (1.7)
Orissa	2 (1.7)
Tripura	2 (1.7)
Sikkim	1 (0.8)
**Location**	
Urban	84 (71.3)
Semi-urban	25 (21.2)
Rural	9 (7.6)
**Highest level of Education**	
Undergraduate	27 (22.9)
Postgraduate	81 (68.6)
Doctorate	10 (8.5)
**Area of Expertise/Practice**	
Musculoskeletal physiotherapy	50 (42.4)
Sports physiotherapy	14 (11.9)
Neurological physiotherapy	23 (19.5)
Pediatric physiotherapy	12 (10.2)
Cardiorespiratory physiotherapy	12 (10.2)
Geriatric physiotherapy	1 (0.8)
Women's health physiotherapy	1 (0.8)
Community physiotherapy	3 (2.5)
Fitness or Yoga	2 (1.7)
**Years of Experience**	
Less than 5	68 (57.6)
6 - 10	20 (16.9)
11 - 20	20 (16.9)
More than 20	10 (8.5)
**Type of Workplace**	
Teaching Hospital	58 (49.2)
General Hospital	28 (23.7)
Private Clinic	32 (27.1)

### TECHNOLOGY USAGE

About half of the respondents (n=61; 51.7%) use computers frequently at work, while most respondents (n=81; 68.7%) use the internet frequently at work. Similarly, just over half of the respondents (n=67; 56.8%) use e-mail frequently at work. Some respondents (n=28; 23.7%) have never used telerehabilitation. Smartphones were the most common electronic devices used for telerehabilitation (n=74; 83.1%), followed by laptops (n=47; 52.8%), desktop PCs (n=12; 13.5%), and tablet computers (n=7; 7.9%). Among those that had used telerehabilitation (n=69), WhatsApp™ video chat (n=24; 33.3%), Zoom™ Cloud Meetings (n=15; 20.8%), Google Meet™ (n=12; 16.7%), and phone calls (n=10; 13.9%) were the most common modes of delivery. Most respondents (n=75; 86.2%) used live sessions with clients for telerehabilitation.

### PERCEPTIONS OF TELEREHABILITATION

An overwhelming majority of respondents (n=109; 92.3%) agreed that ICT has a potential role in healthcare delivery. Most respondents (n=77; 65.3%) agreed that telerehabilitation could be used to manage physical impairments. A slightly larger percentage of respondents (n=86; 72.9%) find telerehabilitation to be a viable approach for the provision of physiotherapy services. Most respondents (n = 91; 77.1%) indicated that telerehabilitation could save time and money. Over half the respondents (n=70; 59.3%) believe that telerehabilitation could reduce the burden on physiotherapists. A minority of respondents (n =39; 33.1%) stated that ICT does not apply to their workplace. Finally, most respondents (n=97; 82.2%) believe telerehabilitation will play an essential role in the profession's future.

### COMFORT WITH TECHNOLOGY

Most respondents (n=75; 63.6%) trust the telerehabilitation technology to work as expected. A majority of respondents (n=103; 87.3%) would be happy to use ICT to provide physiotherapy, including patient education. Similarly, most respondents (n=110; 93.2%) were comfortable using ICT for the storage and retrieval of patient data. Most respondents (n=75; 63.6%) believe that their culture and social norms do not prohibit the use of telerehabilitation.

### WILLINGNESS TO USE TELEREHABILITATION

Based on their current conditions, most respondents felt that telerehabilitation is appropriate for use in their workplace (n=99; 83.9%) and would be happy to use telerehabilitation to deliver physiotherapy (n=84; 71.2%). Most respondents believe that their colleagues would be willing to use telerehabilitation (n=93; 78.8%) and would be happy to recommend telerehabilitation to other physiotherapists (n=88; 74.6%).

### BARRIERS TO USING TELEREHABILITATION

The most common barriers identified by respondents were a lack of training in telerehabilitation practice (n=64; 54.2%) and a lack of connection between ICT experts and clinicians (n=62; 52.5%). Other important barriers were data privacy concerns (n=47; 39.8%), lack of user-friendly software (n=41; 34.7%), perceived lack of clinical utility (n=42; 35.6%), perceived increase in workload (n=25; 21.2%), negative attitudes of staff involved (n=23; 19.5%), and the cost of equipment (n=14; 11.9%). A few respondents (n=16; 13.6%) also mentioned a lack of awareness about telerehabilitation in society, internet connectivity issues, a lack of personal contact/touch, poor patient compliance, low technology literacy, and increased stress caused by explaining and delivering therapy through telerehabilitation as barriers.

**Figure 1:**
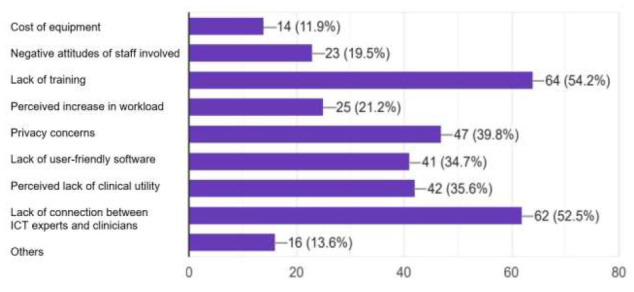
Barriers to Telerehabilitation Usage Identified by Respondents

## DISCUSSION

### PERCEPTIONS OF TELEREHABILITATION

Overall, respondents expressed a positive perception of telerehabilitation. This finding is similar to a survey conducted among physiotherapists in Kuwait (Albahrouh & Buabbas, 2021). Most respondents agree that telerehabilitation will play an essential role in the future of the profession. With the ongoing health crisis resulting from the COVID-19 pandemic, more research is being conducted in telerehabilitation applications to deliver physiotherapy. Although some respondents found ICT inapplicable to their workplaces, most did agree that ICT has a vital role in healthcare delivery. An increase in digital literacy among physiotherapists will ensure easier adoption of telerehabilitation technologies. Clinical trials have specifically approached telerehabilitation from a perspective of increased efficiency in healthcare delivery and the economic impact of telerehabilitation (Seron et al., 2021).

### COMFORT WITH TECHNOLOGY

Most participants trust telerehabilitation technology to work as expected. This might be due to most participants being under 35 years of age and thus are more accustomed to using technology in daily life (Olson et al., 2011). Respondents were happy to use ICT for providing physiotherapy, including patient education. They were comfortable in using ICT for data storage and retrieval. Data privacy concerns on internet usage are gaining more attention, but this was not evident from the results of this survey. Telerehabilitation practitioners and their patients must be aware of data security measures while using any given telerehabilitation system (Hall & Mcgraw, 2014). Most agreed that their cultural and social norms were not prohibitive of telerehabilitation. This might suggest that Indian physiotherapists are receptive to more innovative telerehabilitation systems, and this untapped potential needs to be met with more research and development.

### WILLINGNESS TO USE TELEREHABILITATION

Telerehabilitation was deemed appropriate in the workplaces of most participants. Yet, most respondents reported that they used telerehabilitation for the first time during the pandemic. Respondents are happy to use telerehabilitation to deliver physiotherapy services. Most physiotherapists in this survey responded that they would happily recommend telerehabilitation to other physiotherapists; this will generate more awareness among colleagues who would have otherwise been hesitant to try out new modes of intervention. Hence, telerehabilitation must be incorporated wherever possible to give patients more choices and physiotherapists more tools to serve them (Cranen et al., 2017).

### BARRIERS TO USING TELEREHABILITATION

Most participants reported that a lack of training is the primary barrier limiting their telerehabilitation usage in clinical practice. Massive open online courses and other accessible educational resources must be developed to train physiotherapists to deliver telerehabilitation safely and effectively (Fioratti et al., 2021).

Another common barrier identified was the lack of connection between ICT professionals and physiotherapists. A multidisciplinary approach to telerehabilitation practice is essential (Leochico, 2020). ICT experts need to be consulted, and physiotherapists need to express their needs to reach a consensus. The collaboration will improve both the delivery and efficacy of therapy provided through telerehabilitation.

The cost of telerehabilitation equipment is a significant barrier, especially in the context of low-middle-income countries with limited resources at their disposal. Existing technology such as smartphones and other telecommunication devices could be repurposed for the use of telerehabilitation in low-resource settings (Leochico et al., 2020).

Data privacy concerns, an evolving subject of discussion, were pointed out by a few participants. Resolution of these concerns will require technology companies and governments on a global scale to agree to ensure data privacy rights for all (Hall & Mcgraw, 2014).

User-friendly software and hardware design will improve the clinical utility of telerehabilitation (Mukaino et al., 2020). This needs to be a crucial area of focus to make telerehabilitation more accessible to a broader population.

## CONCLUSION

Physiotherapists working in India have positively perceived telerehabilitation usage during the ongoing COVID-19 pandemic and are willing to utilize such services in clinical practice. This survey identified several significant barriers that need to be addressed to ensure the universal telerehabilitation practice by physiotherapists in India.
